# Meningococcemia in a Boy with Dense Deposit Disease Receiving the C5 Complement Inhibitor Ravulizumab: A Case Report

**DOI:** 10.5811/cpcem.52835

**Published:** 2026-04-07

**Authors:** Andrew J. Gonedes, Alexandra Martinez, Allan M. Greissman, Hanan Atia, Eric Boccio

**Affiliations:** * Memorial Healthcare System, Department of Emergency Medicine, Hollywood, Florida; † Florida International University, Herbert Wertheim College of Medicine, Miami, Florida; ‡ Joe DiMaggio Children’s Hospital, Department of Pediatric Critical Care Medicine, Hollywood, Florida; § Mount Sinai Medical Center of Florida, Department of Emergency Medicine Miami Beach, Florida

**Keywords:** dense deposit disease, meningococcemia, C5 complement inhibitor, ravulizumab, case report

## Abstract

**Introduction:**

Dense deposit disease, also known as C3 glomerulopathy, is a rare renal disorder caused by abnormal complement deposition in the glomerular basement membrane. Patients often require long-term immunosuppressive therapy and, in some cases, complement inhibitors such as ravulizumab. While effective at limiting renal damage, complement blockade significantly increases susceptibility to invasive infections from encapsulated bacteria, particularly *Neisseria meningitidis*. Despite immunization and antimicrobial prophylaxis, these patients remain incompletely protected. We describe a case of meningococcemia in a fully vaccinated adolescent with dense deposit disease on ravulizumab therapy.

**Case Report:**

A 17-year-old male with a history of dense deposit disease on mycophenolate mofetil and ravulizumab presented to the pediatric emergency department with fever, vomiting, altered mental status, and a rapidly evolving petechial-purpuric rash. He was fully immunized with both meningococcal conjugate and serogroup B vaccines. Initial evaluation revealed fever, hypotension, and altered level of consciousness. Laboratory studies showed leukocytosis, elevated inflammatory markers, and blood cultures subsequently confirmed *N. meningitidis*. Empiric ceftriaxone and vancomycin were initiated, later narrowed to ceftriaxone. Supportive management included intravenous (IV) fluids, vasopressors, IV immunoglobulin, and dexamethasone. The patient demonstrated rapid improvement, with resolution of hemodynamic instability and normalization of kidney function. He was discharged on hospital day eight with prophylactic penicillin and close outpatient follow-up.

**Conclusion:**

This case underscores the risk associated with complement inhibition, even in fully vaccinated individuals. Clinicians must maintain high vigilance for meningococcal disease in immunocompromised patients and initiate early aggressive therapy to optimize outcomes.

## INTRODUCTION

Dense deposit disease (DDD), also known as C3 glomerulopathy, is a rare kidney disease caused by abnormal deposition of complement component C3 in the glomerular basement membrane. It can result in significant renal dysfunction and has a poor prognosis if left untreated. It is typically treated with immunosuppressive therapy. In certain cases, complement inhibitors such as ravulizumab are used to modulate the immune system and prevent further kidney injury. However, these therapies leave patients vulnerable to encapsulated organisms such as *Neisseria meningitidis*, which can cause severe and life-threatening conditions such as meningococcal meningitis and meningococcemia. Despite vaccinations and prophylactic antimicrobials, these patients remain incompletely protected. We present the case of a 17-year-old male with a history of DDD and taking the C5 complement inhibitor ravulizumab who developed meningococcemia despite having received both the meningococcal conjugate vaccine (MCV4) and serogroup B vaccine (MenB).

## CASE REPORT

A 17-year-old male with past medical history significant for DDD presented to the pediatric emergency department (ED) with fever and altered mental status. The initial diagnosis of DDD was made when the patient was eight years of age following workup that revealed proteinuria, hematuria, and progressive renal impairment. Genetic testing was confirmatory for the disease, and the patient was started on immunosuppressive therapy which included mycophenolate mofetil monotherapy initially and mycophenolate mofetil and rovelizumab dual therapy subsequently. At the time of ED presentation, the patient was up-to-date with age- and risk-specific vaccinations including MCV4 and MenB.

One week prior to presentation, the patient developed a fever, headache, nausea, and vomiting. Upon arrival to the pediatric ED, the patient was lethargic and altered, unable to respond to questions or participate in the physical examination. Initial ED vital signs were remarkable for hypotension (blood pressure 90/54 millimeters (mm) of mercury); tachycardia (heart rate 164 beats per minute); tachypnea (respiratory rate 20 breaths per minute); and fever (temperature 39.4 °C, oral). The patient was able to localize noxious stimuli, and the initial Glasgow Coma Scale score was seven. Physical examination revealed nuchal rigidity and a positive Brudzinski sign. A petechial rash developed, which rapidly coalesced into larger purpuric lesions across the chest, upper and lower extremities, and bilateral hands (Image).

Given the patient’s altered mental status, rapid sequence intubation was considered for airway protection; however, the procedure was deferred initially until hemodynamics could be optimized. Initial laboratory workup revealed the following: leukopenia, 3,600 cells per microliter (μL) [reference range: 4,500–11,000 cells/μL]; elevated C-reactive protein, 4.99 milligrams (mg) per deciliter (dL) [< 0.9 mg/dL]; elevated procalcitonin, > 200 nanograms (ng) per μL [< 0.05 ng/μL]; elevated serum creatinine, 1.99 mg/dL [0.7–1.3 mg/dL]; anion gap metabolic acidosis, 20 milliequivalents (mEq) per liter (L) [8–12 mEq/L]; and decreased serum bicarbonate, 12 mEq/L [22–29 mEq/L]. Peripheral blood cultures were obtained. The performance of a lumbar puncture in the ED was deferred due to the patient’s hemodynamic instability.

*CPC-EM Capsule*

What do we already know about this clinical entity?

*Meningococcemia is a rapidly progressive and life-threatening infection with greatly increased risk in patients receiving C5 complement inhibitors*.

What makes this presentation of disease reportable?

*We present a case of meningococcemia in a fully vaccinated adolescent on ravulizumab, illustrating meningococcal risk despite vaccination and prophylaxis*.

What is the major learning point?

*Vaccination does not fully protect patients on C5 inhibitors, making early recognition and prompt aggressive treatment essential for patient survival*.

How might this improve emergency medicine practice?

*High clinical suspicion and timely initiation of empiric therapy for meningococcemia is paramount in immunocompromised patients, even if vaccinated*.

Given the high clinical suspicion for meningococcemia, aggressive fluid resuscitation and empiric antimicrobial therapy consisting of ceftriaxone and vancomycin was initiated. Additionally, intravenous immunoglobulin (IVIG) was administered to support the immune response in the setting of immunosuppressive therapy. Following aggressive fluid resuscitation, the patient remained hypotensive, and dexamethasone was given as adjunctive therapy for refractory septic shock. Blood cultures grew *N. meningitidis*, and the antibiotic regimen was narrowed to ceftriaxone. The patient was admitted to the pediatric intensive care unit where vasopressors were initiated, and infectious disease and nephrology services were consulted.

Within 72 hours of initiating targeted therapy, the patient’s clinical condition began to improve. The patient defervesced and his mental status improved, returning to baseline on hospital day three. Following stabilization of his hemodynamics, vasopressors were discontinued. Repeat laboratory test results revealed declining inflammatory markers and normalization of kidney function. Doses of mycophenolate and ravulizumab were held during the entirety of the hospital length of stay. On hospital day eight, the patient was discharged home, and prophylactic penicillin V was added to his medication regimen. Outpatient follow-up with nephrology and infectious diseases were arranged, and the patient was scheduled to receive vaccine boosters at his next outpatient visit.

## DISCUSSION

Meningococcal meningitis is a serious bacterial infection that causes inflammation of the membranes surrounding the brain and spinal cord. Symptoms may include fever, headache, nuchal rigidity, nausea, vomiting, photophobia, and altered mental status. In some cases, *N. meningitidis* may infect the bloodstream, a condition called meningococcemia. In the post-*Haemophilus influenzae* type b era, the presence of rash in cases of meningococcemia has declined; however, when present, it is often pathognomonic.^1^–^2^ The rash typically starts as petechiae – red or purple lesions caused by bleeding capillaries under the skin - < 3 mm in diameter and may rapidly coalesce into larger purpura (3–10 mm in diameter) or ecchymoses (> 10 mm in diameter).

In the most severe cases, the pro-inflammatory response triggered by the release of bacterial endotoxins may result in a hypercoagulable state known as disseminated intravascular coagulation that results in overactivation of the clotting cascade in the microvasculature. This results in the consumption of platelets and clotting factors leading to a hypocoagulable state that may precipitate bleeding across various sites including the skin, nose, mouth, and solid organs. The skin rashes associated with meningococcemia are a sign of severe systemic involvement and imminent multisystem organ failure and indicates a poor prognosis if not treated promptly.^3^–^4^ According to the U.S. Centers for Disease Control and Prevention (CDC) Health Alert Network, meningococcal disease carries a case-fatality rate of 10–15%. An increase in invasive meningococcal disease cases has been noted with 143 cases reported in the first quarter of the 2024 calendar year and 422 cases reported in calendar year 2023.^5^

*Neisseria meningitidis* is a gram-negative bacterium that can cause meningococcal meningitis and meningococcemia in susceptible individuals. The bacterium’s polysaccharide capsule contributes to its virulence interfering with phagocytosis and antibody-mediated killing. In immunocompromised patients, particularly those on complement inhibitors such as ravulizumab, the ability to clear encapsulated organisms such as *Neisseria meningitidis*, *Streptococcus pneumoniae*, and *Haemophilus influenzae* is significantly impaired.^6^ Ravulizumab inhibits complement component C5 which is cleaved by C5 convertase into C5a and C5b thus preventing formation of the membrane attack complex which is essential for the perforation and osmotic cell lysis of invading pathogens.^7^ According to the CDC, the risk of meningococcal disease is up to 2,000 times greater for people receiving complement inhibitors compared to healthy controls.

The U.S. Food and Drug Administration-approved prescribing information for complement inhibitors includes a black box warning for increased meningococcal disease risk and recommends administration of meningococcal vaccines to patients receiving complement inhibitors.^8^ Despite appropriate vaccination and antimicrobial prophylaxis, substantial risk remains, as CDC data suggest meningococcal vaccines likely provide incomplete protection against invasive meningococcal disease in patients receiving C5 inhibitors.^9^ The patient had received both the MCV4 and MenB vaccines. While nongroupable *N. meningitidis* caused most infections identified in patients receiving eculizumab, experts believe that patients on other C5 inhibitors may be susceptible to similar strains.^10^ Of note, in addition to ravulizumab, the patient was also taking mycophenolate mofetil, which likely further suppressed the immune system’s proliferation of T and B cell lymphocytes.

Given the potential for rapid deterioration in immunocompromised individuals, management of meningococcal meningitis requires early identification, prompt initiation of appropriate antibiotics, and continued supportive care. Empiric therapy for suspected meningococcal disease should include an extended-spectrum cephalosporin, such as cefotaxime or ceftriaxone. Once the diagnosis is established, definitive treatment can be continued with an extended-spectrum cephalosporin, or, alternatively, if susceptibility to penicillin is confirmed, treatment can be switched to penicillin G or ampicillin.^11^–^12^ Adjunctive therapies, such as corticosteroids to reduce inflammation and IVIG to support the immune system, may also be beneficial in improving outcomes.^13^ Preventive strategies, including vaccination and prophylactic antibiotics, are staples of care in at-risk populations.^14^

## CONCLUSION

This case highlights the increased risk of invasive infections in pediatric patients on immunosuppressants. Despite being fully vaccinated, the patient developed meningococcemia, underscoring the limitations of vaccinations in providing complete protection in patients on C5 complement inhibitors such as ravulizumab. The patient presented clinically with the characteristic petechial rash, which rapidly progressed to purpura, pathognomonic for meningococcemia when other clinical symptoms of meningococcal meningitis are present. Early identification, prompt treatment with empiric and then targeted antimicrobial therapy, and aggressive resuscitation were essential to the patient’s survival and recovery. This case emphasizes the importance of initiating preventive strategies while recognizing their limitations, counseling patients about vigilant surveillance and monitoring, and maintaining a high index of clinical suspicion for meningococcal disease in patients who receive complement inhibitors.

## Figures and Tables

**Image:**
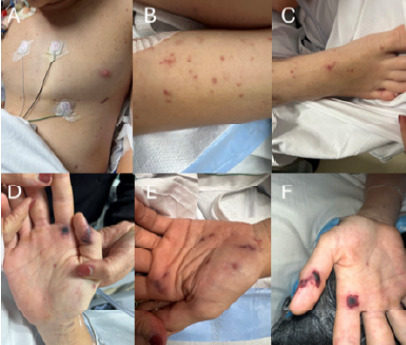
Lesions ranging from small red and purple flat petechia (< 3 millimeters (mm) in diameter) to large red and purple purpura (3–10 mm in diameter) noted across the skin of the chest (A), upper extremities (B), lower extremities (C), and palms of the bilateral hands (D–F) concerning for meningococcemia.
